# Knoop Hardness of Self-Etch Adhesives Applied on Superficial and Deep Dentin

**DOI:** 10.30476/DENTJODS.2019.77805.0

**Published:** 2020-03

**Authors:** Maryam Firouzmandi, Shideh Khashaei

**Affiliations:** 1 Dept. of Operative Dentistry, School of Dentistry, Shiraz University of Medical Sciences, Shiraz, Iran; 2 Graduated Dental Student, Student Research Committee, School of Dentistry, Shiraz University of Medical Science, Shiraz, Iran

**Keywords:** Self-etch adhesives, Polymerization, Hardness, Dentin

## Abstract

**Statement of the Problem:**

Low pH of self-etch adhesives might cause suboptimal polymerization.

**Purpose:**

This study aimed to evaluate the effect of dentin depth (deep and superficial) on polymerization efficacy of two self-etch adhesives, with different pH by means of Knoop hardness test.

**Materials and Method:**

In this in vitro study, sixty sound molars were used to prepare 30 superficial dentin and 30 deep dentin specimens. Dentin specimens of each depth were randomly distributed into two equal subgroups (N=15) and bonded by either Adper Prompt L-Pop (strong self-etch adhesive) or Adper Easy Bond (mild self-etch adhesive). Knoop hardness test was employed to evaluate degree of cross-linking of the adhesives. Data were analyzed with SPSS 16, using two-way ANOVA to compare mean hardness values of the study groups (*p*< 0.05).

**Results:**

There was no interaction effect between dentin depth and the type of adhesive (*p*= 0.36). Regardless of dentin depth, hardness of Adper Easy Bond was significantly higher than that of Adper Prompt L-pop (*p*< 0.001). Moreover, both the adhesives showed higher hardness when bonded to superficial dentin compared to deep dentin (*p*< 0.001).

**Conclusion:**

Degree of cross-linking of the self-etch adhesive with mild acidity was more than that of the strong self-etch adhesive after light-curing. Surface hardness of both adhesives was higher on superficial dentin compared to deep dentin.

## Introduction

Tooth-colored restorative materials accompanied by adhesive techniques are increasingly demanded by patients. The complexity and sensitivity of placement techniques associated with adhesive systems are their main disadvantages. The most conventional form of the adhesives involves three application steps. These steps include etching and rinsing, priming and bonding [ 1 ]. A trend for simplification of adhesives systems has begun after the emergence of priming and bonding steps. In this way self-etch approach was introduced in 1994, consisting of two or one application step(s) [ 1 ]. The elimination of etch-and-rinse phase not only reduced clinical application time but also made them more user friendly and less technique-sensitive [ 2 ]. 

The interaction of self-etch adhesives (SEAs) with tooth substrate is through simultaneous dissolution of hydroxyapatite (HAP) crystals from tooth structure and infiltration of resin into the created spaces [ 1 ]. The concurrency of etching and resin infiltration lowers the risk of discrepancy between both processes [ 3 - 4 ]. This phenomenon becomes possible through the unique composition of SEAs. They incorporate unsaturated, potentially polymerizable acidic monomers [ 5 ]. The etching capacity of these monomers is due to their phosphoric ester or carboxylic acid groups. However, the degree of interaction with tooth substrate depends on their aggressiveness. In this regard, they can be subdivided into strong (pH<1), intermediate (1<pH<2) and mild (pH>2) [ 3 ]. The chemical reaction/interaction of HAP with functional acidic monomers is substantial in terms of bond strength [ 6 ], interfacial bonding morphology [ 1 ], nanoleakage [ 7 ] and monomer conversion [ 7 ]. 

Polymerization is important in terms of physico-mechanical strength of the adhesive [ 4 ]. In simplified adhesives, the degree of conversion was shown to be low [ 8 ]. This can cause low mechanical properties, higher permeability [ 8 ], more water sorption [ 9 ], more nanoleakage [ 9 ], degradation of the bonding interface [ 10 ], more leaching out of residual unpolymerized monomers and lower biocompatibility [ 11 ]. Polymerization is inhibited by several factors such as the presence of oxygen [ 12 ], presence of intrinsic water from dentin and solvents [ 13 ]. Another reason postulated for incomplete conversion of SEAs is the interaction of acidic monomers with amines of the photoinitiator system [ 14 ]. 

Previous studies have shown that the type of substrate has a great influence on polymerization of strong SEAs. Enhancement of polymerization was achieved through their interaction with dentin or enamel [ 5 , 14 - 16 ]. It was concluded that minerals from enamel or dentin buffer the acidic monomers. In the absence of minerals these monomers consume amines of the photoinitiator system. Consequently, the polymerization reaction is impaired [ 14 ]. 

Dentin is a complex biomaterial made up of four structural components: tubules, peritubular dentin, intertubular dentin, and dentinal fluid [ 17 ]. There is a difference in contribution of each component to dentin composition between deep and superficial dentin [ 18 ]. Therefore, the properties of the substrate available for bonding vary with different regions [ 18 ]. Bonding to deep dentin is more challenging compared to superficial dentin. This is due to the lower amount of intertubular dentin and larger diameter and density of tubules in deeper regions [ 18 ]. 

The above-mentioned issues bring into mind the possibility of the effect of dentin mineral density, which differs depending on the effect of dentin depth on polymerization of SEAs. The present study investigated the effect of dentin depth on polymerization of mild and strong SEAs using microhardness test. It has been accepted that surface hardness can be an indicator of degree of conversion of dental resins [ 19 ]. The null hypothesis to be tested was that the acidity of the adhesive and dentin depth does not influence hardness of the cured adhesive. 

## Materials and Method

Sixty human third molars (age 18-35) extracted for surgical reasons were used in this study. The teeth were sound, caries-free and without any restorations. The study protocol was approved by the Ethic Committee of Shiraz University of Medical Sciences. The teeth were stored in 0.5% chloramine solution at 4°C and used within one month after extraction. 

### Preparation of specimens 

In order to prepare superficial dentin specimens (SD), the occlusal surface of 30 teeth were flattened with a diamond saw (Isomet; Buehler, Lake Bluff, IL, USA). Then it was grounded with 200 grit abrasive paper until the entire enamel was removed and the occlusal dentin was exposed. The roots were also removed 2 mm below the cementoenamel (CEJ) junction.

To obtain deep dentin (DD) specimens, in the remaining 30 teeth two cuts parallel with occlusal surface of the teeth, one 2 mm above the CEJ and the other 2 mm below the CEJ, were made using a diamond saw (Isomet; Buehler, Lake Bluff, IL, USA). Then the occlusal surface of the specimens was grounded with 400-grit silicon carbide papers to reach a remaining dentin thickness of 0.9±0.1 mm. The thickness of remaining dentin was measured manually with a dental gauge caliper (stainless steel Iwanson caliper, 0-10mm, Neuhausen, Germany) in the areas corresponding to the hieghst pulp horn.

Superficial and deep dentin specimens were then sectioned prependicuar to the abraded surface to obtain an equal-sized surface area of 5×5 mm and were further abraded with 600-grit silicon carbide paper to create a uniform smear layer. All the preparations were carried out under water cooling.

### Experimental groups

The specimens were divided into four groups (n=15). In the group SD/APLP, the specimens had superficial dentin, which were bonded with Adper Prompt L-pop adhesive system. In the group SD/AEB, the specimens had superficial dentin, which were bonded with Adper Easy Bond adhesive system. In the group DD/APLP, the specimens had deep dentin, which were bonded with Adper Prompt L-pop adhesive system. In the group DD/AEB, the specimens had deep dentin, which were bonded with Adper Easy Bond adhesive system. Detailed information about the adhesives and their application method is provided in Table 1.

**Table1:** The composition of adhesives used in this study

Adhesive	Composition	Lot-number	Application mode
Adper Prompt L-pop (3M ESPE, Neuss, Germany)	Methacrylated phosphoric esters, Bis-GMA, 2-Hydroxyethyl methacrylate (HEMA), Polyalcenoic acid, Camphorquinone, Stabilizers, Water	602032	● rubbing for 15s
			● gentle air drying
			● applying second coat and gentle air drying
			● light-curing for 10s
Adper Easy Bond (3M ESPE, Neuss, Germany)	Methacrylated phosphoric esters, Bis-GMA, 2-Hydroxyethyl methacrylate (HEMA), Polyalcenoic acid, 1,6 hexanediol dimethacrylate, silica filler, Camphorquinone, Stabilizers, Water, ethanol	590946	rubbing for 20s
			● gentle air drying
			● light-curing for 10s

The output intensity of light-emitting diode (LED) curing light (Blue LEX 1200W, MONITEX, San-Chong City, Taipei, Taiwan) was measured with a radiometer to be 900-1000 mw/cm^2^ before curing each specimen. The surfaces were checked to ensure uniform distribution of adhesive and a glass coverslip was placed on the top of the adhesive layer to create a flat surface, and avoid contact with the atmospheric oxygen during light activation. After the bonding procedures were completed, the specimens were kept in a dry dark container at 37°C because the adhesive layer plasticizes in contact with moisture. The specimens were submitted to hardness testing after 15 minutes to standardize the effect of post curing polymerization.

### Measurement of hardness 

The specimens’ hardness was measured with Shimadzu HMV-2 hardness tester (Shimadzu Corporation, Kyoto, Japan), equipped with Knoop indenter at 0.245-N load and 10 seconds of dwell time. Five indentations were prepared on each specimen. In deep dentin specimens, the indentations were made near the sites corresponding to the pulp horns, where the remaining dentin thickness was lower.

The dimensions of the indentations were determined by examining the surface with an optical microscope (40×) and expressed as the Knoop hardness number. The mean value of the five indentations was reported as the hardness of each specimen.

### Statistical analysis

Knoop hardness numbers determined for the groups were analyzed by two-way ANOVA. Dentin depth and type of adhesive were the main factors and the significance level was set at α=0.05.

## Results

The means and standard deviations of Knoop hardness of the two SEAs applied on superficial and deep dentin are summarized in Table 2. The results of two-way ANOVA showed no interaction effect between dentin depth and the type of adhesive (*p*= 0.36). This means that the type of the adhesive did not affect its degree of conversion on different dentin depth. Regardless of dentin depth, hardness of Adper Easy Bond was significantly higher than Adper Prompt L-pop (*p*< 0.001). Moreover, both of the adhesives showed higher hardness when bonded to superficial dentin compared to deep dentin (*p*< 0.001). 

**Table2:** Mean knoop hardness numbers ± standard deviations of study groups and statistical significance

Dentin depth	Adper Prompt L-Pop	Adper Easy Bond
Superficial dentin	10.24±2.2 ^A,a^	14.3±2.07 ^A,b^
Deep dentin	8.98±1.2 ^B,a^	12.02±1.3 ^B,b^

Groups with different upper case letters are statistically different in the columns. Groups with different lower case letters are statistically different in the rows (*p*< 0.001).

## Discussion

The null hypothesis was rejected because hardness of mild SEA used in this study was higher than the strong SEA, and both adhesives were harder on superficial dentin. According to the results of the present study, hardness of AEB was higher than APLA both on deep and superficial dentin, which can be translated into higher degree of conversion of AEB. Other studies also confirmed more complete polymerization in AEB [ 5 ]. Although APLP has been reported to show favorable adhesion to both enamel and dentin [ 20 ], there are some concerns about its polymerization. Despite similarity in chemical composition between AEB and APLP, there are minor differences which favor the distinct polymerization behavior. One of the factors influencing polymerization of monomers is the viscosity. According to gel effect or Trommsdorff-Norrish phenomenon, polymerization rate increases at high viscosity [ 21 - 22 ]. AEB contains more Bis-GMA than APLP [ 14 ]. Bis-GMA is a viscous molecule with high molecular weight, and can enhance polymerization by generating cross-linked three-dimensional resin network [ 23 ].

AEB is a filled adhesive with silica fillers [ 14 ]. Fillers increase viscosity of the adhesive. Additionally fillers may strengthen the adhesive layer. Filled adhesives create thicker layers after air drying, preventing incomplete polymerization due to oxygen inhibition [ 4 ]. However, in the present study the effect of oxygen was eliminated by curing the adhesive through a glass slip in contact with adhesive surface.

The other factor influencing adhesive polymerization is incomplete evaporation of solvents [ 2 ]. The only solvent present in APLP is water but AEB contains ethanol as a co-solvent for water [ 14 ]. Water hardly evaporates due to high boiling temperature, low vapor pressure and presence of HEMA. HEMA induces more water retention by creating hydrogel structures [ 4 ]. This can cause adhesive plasticization and polymerization hindrance [ 24 ]. Presence of ethanol in conjunction with water improves solvent evaporation from the adhesive than pure water [ 4 ]. 

The results of the present study showed that dentin depth influenced hardness of both adhesives regardless of their pH. Hardness of both adhesives on superficial dentin was higher than that on deep dentin. Etch-and-rinse adhesive systems alter dentin structure, surface morphology and physical properties to a greater extent. In contrast, in self-etching concept, the adhesive diffuses through the smear layer into intact dentin. It was assumed that the mechanical properties of this adhesive layer were dependent on the hardness of the underlying dentin substrate [ 25 ]. Knoop hardness of superficial dentin has been shown to be significantly higher than deep dentin [ 25 ]. Knoop hardness measurements are sensitive to surface effects and texture. This difference reflects variation in tubule density and the amount of dentin mineral dispersed within the collagen matrix in different locations [ 26 ]. Previous studies have shown that the type of substrate greatly influences polymerization of strong SEA such as APLP [ 5 , 14 , 27 ]. The photoinitiator system in both adhesives is composed of camphorquinone and amine accelerator. Nitrogen atom of amine molecule goes through an interaction with acidic functional monomer. This event is the basis for polymerization system retardation in SEAs with high concentration of acidic monomers [ 28 ]. The aforementioned interaction might proceed to a second step in the presence of external additives such as HAP. This compound decomposes amine-acid complex and releases amine co-initiator to trigger spontaneous polymerization [ 28 - 29 ]. Meanwhile HAP serves as a chemical interaction target to promote stable chemical bonds with acidic monomer to enhance bond strength and durability [ 29 ]. As a mild SEA, AEB showed high polymerization degree independent of the type of substrate. In our study there was no interaction effect between the type of the adhesive and the type of the substrate (SD or DD). Despite the difference between the mineral content of superficial and deep dentin, polymerization of AEB and APLP on both substrates was similar. It seems that even the lower amount of minerals in deep dentin was sufficient to neutralize the acidic monomers of highly acidic APLP. Therefore, the initiator system was not invaded by these monomers. In addition, based on the manufacturer’s instructions, both adhesives were applied with a rubbing motion. Adhesive application with agitation on the surface might help in solvent evaporation. At the same time this method of application carries the acidic monomers inside the smear layer, deep into dentin causing more efficient etching, monomer diffusion, increased hybrid layer thickness [ 30 ], and removal of entrapped air and bubbles [ 31 ]. Previous research found this method of application to increase dentin bond strength [ 14 ] and polymerization efficacy of APLP [ 5 ]. However, the mode of application did not influence AEB polymerization [ 5 ]. 

After light curing, the specimens were kept in a dark container and hardness was measured after 15 minutes. A study showed that DC of a model SEA with trimethylbenzoyl-diphenylphosphine oxide (TPO) photoinitiator reached the maximum value (100%) 125 minutes after light-curing [ 29 ]. They proposed that HAP released from etching of tooth structure can trigger a self-curing phenomenon, so-called base-triggered radical polymerization, via the amine-acid complex (BT-RPAAC) [ 29 ]. This phenomenon also could happen in SEAs with camphorquinone initiator [ 29 ]. Accordingly, SEAs used in our study could promote BT-RPAAC. Therefore, it is recommended that the effect of varying percentages of HAP in deep and superficial dentin on self-curing phenomenon be studied by extending the time lag between application of the adhesive and measurement of hardness in future research.

Raman spectroscopy has the ability to evaluate the in situ polymerization behavior but Knoop hardness test used in this study was simply an overall estimation of degree of cross-linking of the adhesive [ 29 ]. For more detailed information, complementary tests are recommended in future studies. Dentin internal wetness caused by outward tubular fluid under pulpal pressure might affect polymerization of adhesives. The effect of pulpal pressure is more pronounced in deep dentin because of larger diameter and density of tubules [ 32 ]. Thus the effect of pulpal pressure on polymerization of adhesives was investigated in a parallel study.

## Conclusion

Adhesion was affected by different factors such as dentin depth and the type of the self-etch adhesive being used. The results showed that bonding of mild self-etch adhesive to superficial dentin promoted higher values for degree of conversion and hardness. The lowest Knoop hardness number was associated with bonding of the strong self-etch adhesive to deep dentin.
